# Effects of a Cognitive-Behavioral Group Counseling Program on Weight Bias Internalization and Psychological Outcomes Among Obese Youth in Mahasarakham University, Thailand

**DOI:** 10.34172/jrhs.2024.155

**Published:** 2024-07-31

**Authors:** Suneerat Yangyuen, Thidarat Somdee, Meihua Yin, Atchara Chaichan, Supattra Keawmuang

**Affiliations:** ^1^Faculty of Public Health, Mahasarakham University, Thailand; ^2^Faculty of Nursing, Mahasarakham University, Thailand

**Keywords:** Weight bias internalization, Cognitive-behavioral, Counseling, Youths, Obesity

## Abstract

**Background:** Weight bias internalization (WBI) is a serious challenge because of its negative impact on psychological consequences. Although the cognitive-behavioral intervention has been applied to reduce WBI, little is known about its effectiveness among Thai obese youth. Thus, this study sought to determine the effects of a cognitive-behavioral group counseling (CBGC) program on WBI and psychological outcomes in obese youths.

**Study Design:** A randomized controlled trial.

**Methods:** Eighty obese youths were randomly allocated to two intervention (n=40) and control (n=40) groups. The intervention group received a CBGC program in three sessions with ten activities, while the control group performed their usual counseling process. Data were collected through standardized interviewers with a structured interview questionnaire. The chi-square test, independent samples t-test, Mann-Whitney U test, repeated measure ANOVA, and multivariate linear regression were applied for data analyses.

**Results:** At the follow-up visit, the intervention group had a significant decrease in the mean of WBI, depression, and perceived stress and an increased mean of self-esteem compared to the control group. After adjusting baseline characteristics and baseline outcome values, the intervention also significantly improved WBI (B=-6.82, *P*<0.001), depression (B=-4.17, *P*<0.001), perceived stress (B=-6.01, *P*<0.001), and self-esteem (B=5.07, *P*<0.001).

**Conclusion:** The CBGC program effectively reduced WBI, depression, and perceived stress while improving self-esteem among obese youths. This study recommends that group counseling programs be employed as part of a counseling process for obese youth who have experienced WBI.

## Background

 Weight bias internalization (WBI) or weight self-stigma is a serious problem, especially among youth with obesity.^1^ WBI occurs when individuals with obesity absorb negative weight-based societal perceptions and stereotypes, leading to self-devaluation concerning weight.^2^ WBI has been associated with negative health consequences (both physical and psychological),^3^ including depression, stress, lowered self-esteem, eating disorders, and reduced physical activity.^2,3^ Additionally, both males and females are vulnerable to WBI because they have reported internalizing body image ideals and shape/weight dissatisfaction, which increased the risk of WBI.^4,5^ However, there are mixed findings in the literature on whether males and females experience differences in WBI. Some studies showed no difference in WBI,^6,7^ while others found that females reported more WBI as compared to males.^4,8^ In Thailand, prior research has revealed that approximately 48.2% of adolescents experienced cyberbullying about their weight.^9^ In addition, Thai youth perceived beauty standards as cultural ideals, and those who were overweight or obese were at higher risk of psychological distress (e.g., depression, stress, and low self-esteem) due to their weight and body size.^10,11^ Therefore, Thai youth may encounter WBI and its adverse psychological outcomes, and psychological interventions are needed to reduce weight stigma.

 Previous studies have shown that psychological interventions designed to reduce WBI are lacking.^2^ However, one of the most effective approaches to reducing WBI is the cognitive-behavioral approach,^12,13^ which includes skills such as the identification of negative automatic thoughts and the connection among thoughts, feelings, and behaviors.^14^ Such cognitive-behavioral interventions have been applied and confirmed to be effective in improving psychological health, reducing negative attitudes toward people with obesity, and decreasing WBI.^12,13,15^ A literature review represented that group-based cognitive-behavioral (GCB) interventions were effective in treating obese adolescents, including cognitive-behavioral group counseling (CBGC). This is an efficient approach that combines cognitive counseling with behavior so that individuals can better express their thoughts and feelings in the group, have interpersonal discussions, participate, and assist each group member to effectively change their behavior.^12,16^ Some studies have demonstrated that GCB interventions for internalized stigma related to psychological problems can help individuals challenge negative beliefs, cope with stigmatizing situations, and build self-efficacy to achieve goals.^12,17^ Thus, applying this approach to reduce WBI may benefit obese adolescents.

 In Thailand, however, psychological interventions designed to reduce WBI remain limited because most cognitive-behavioral intervention studies have focused on group-based interventions to improve psychological problems in youth.^18,19^ Accordingly, this study sought to determine the effects of a CBGC program on WBI and its negative psychological consequences. This intervention was hypothesized to reduce WBI and improve psychological outcomes, which may help obese youth cope with their WBI.

## Methods

###  Study design and settings

 This randomized controlled trial was conducted among obese youths who attended services at the counseling clinics of a university in northeastern Thailand from May to October 2023.

###  Study participants 

 The sample size was calculated^20^ with the formula n = (Zα + Z_1-β_)^2^ *2 * σ^2^/(µ_1_-µ_2_)^2^. According to a previous study,^13^ considering the mean difference (µ_1_–µ_2_) of 4.4 in the WBI score between the experimental and control groups after the intervention, with a standard deviation (σ) of 6.6, a 95% confidence interval, 80% test power, and a 30% dropout factor, the total sample size required for each group was 40. Further, the total minimum sample size for the study was 80. The inclusion criteria for participants were (1) a body mass index (BMI) of ≥ 25 kg/m^2^,^21^ (2) Thai nationality, (3) participation in counseling services for weight or body size, (4) age range of 18–22 years, (5) lack of communication problems, and (6) willingness to participate in the study. On the other hand, the exclusion criteria included a lack of tendency to participate, absence from the counseling program for more than one session, incomplete responses to interview questions, a history of severe mental disorders, or a leave of absence during the study.

###  Recruitment and randomization 

**Figure 1:**
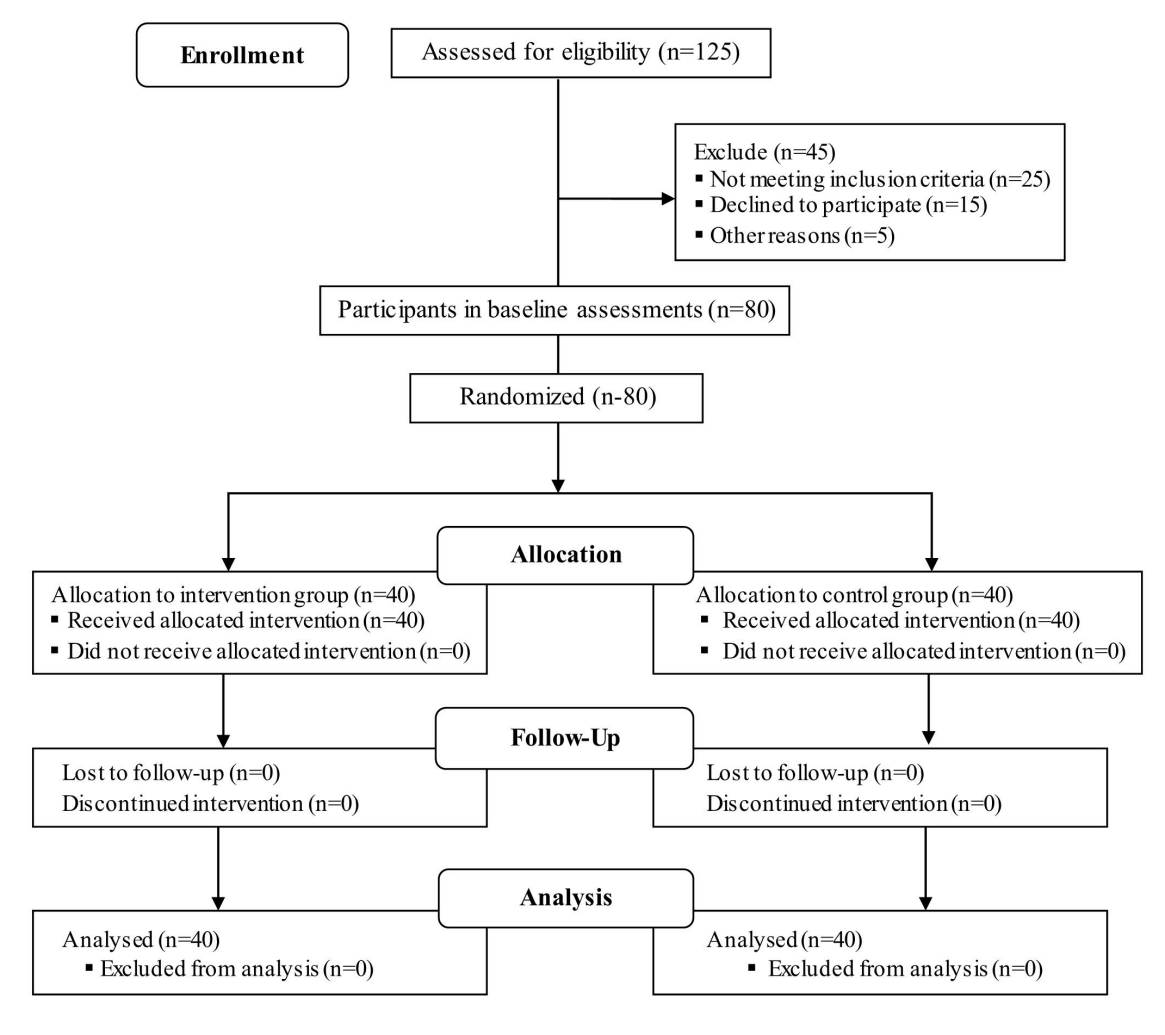
Overview of recruitment and group assignment

 The study recruited students attending university counseling clinics concerning weight or body size problems. They were examined based on the inclusion criteria, and eligible individuals were identified accordingly. The students who met the criteria were allocated, in a 1:1 ratio, to either the control or intervention group by computer-generated random numbers. Initially, 125 students were assessed for participation in this study. However, 25 did not meet the inclusion criteria, 15 were not willing to participate, and five provided incomplete responses to the baseline interview. Thus, 80 students entered the study and were randomly assigned to the intervention and control groups, each containing 40 students (Figure 1). The randomization was conducted by an independent statistician who was not involved in the outcome assessments. All researchers who performed the outcome assessments and analyses were blinded to the group allocations. The randomization took place after the baseline measurements were completed to ensure allocation concealment.

###  Data collection tool and technique 

 The primary outcome was the change in the WBI score from baseline to after the intervention and at the follow-up visit (4 weeks after the intervention). The secondary outcomes were changes in the scores of perceived stress, depressive symptoms, and self-esteem. The data were collected by trained interviewers. The participants were interviewed in a private room with structured interview questionnaires consisting of three parts as follows:

*Part 1:* Sociodemographic variables comprised age, gender, monthly household income, peers with obesity, body image satisfaction, BMI, and experiences of weight stigma. The BMI was calculated as weight (kg) divided by height (m^2^). A portable height and weight meter was used to gauge the students’ height and weight. Their weight status was classified into underweight (BMI < 18.5 kg/m^2^), normal weight (BMI: 18.5–22.9 kg/m^2^), overweight (BMI: 23–24.9 kg/m^2^), or obese (BMI ≥ 25 kg/m^2^) with the Asia-Pacific BMI classification.^21^ The experience of weight stigma was assessed using the Brief Stigmatizing Situation Inventory (SSI-B),^22^ which consists of 10 items rated on a 10-point scale, from 0 (never) to 9 (daily; Cronbach’s alpha was 0.82, indicating good internal consistency). The content validity index (CVI) was 0.8. Higher scores indicate greater exposure to stigmatizing experiences.

*Part 2:* The WBI was measured by the Modified Weight Bias Internalization Scale (WBIS-M),^23^ which assesses the extent to which students blame themselves for stigma and apply negative weight-based stereotypes to themselves. This is a 10-item tool scored on a 7-point Likert-type scale, ranging from 1 (strongly disagree) to 7 (strongly agree; Cronbach’s alpha was 0.83, implying good internal consistency, and the CVI was 1.0). Higher scores represent a higher WBI.

*Part 3:* The measured psychological factors were depression, perceived stress, and self-esteem. The 21-item Beck Depression Inventory-II^24^ was utilized to assess depression symptoms. The tool uses a 4-point scale, ranging from 0 to 3. The total score ranges from 0 to 63, with higher scores indicating greater severity of depression symptoms. The scale showed good internal consistency (Cronbach’s alpha was 0.84, and the CVI was 1.0). Perceived stress was assessed using the Thai version of the Perceived Stress Scale-10, translated and validated by Wongpakaran and Wongpakaran.^25^ This is a 10-item self-report tool rated on a 5-point Likert-type scale, ranging from 0 (never) to 4 (very often). The total score ranges from 0 to 40, with higher scores demonstrating greater perceived stress (Cronbach’s alpha was 0.88, indicating good internal consistency, and the CVI was 0.8). Self-esteem was measured using the Rosenberg Self-Esteem Scale.^26,27^ This is a 10-item questionnaire with a 4-point Likert-type scale, ranging from 1 (strongly disagree) to 4 (strongly agree). Total scores range from 10 to 40, with higher scores indicating higher self-esteem (Cronbach’s alpha was 0.82, representing good internal consistency, and the CVI was 0.8).

###  Intervention

 After a literature review and discussion with specialists in health education, health behavior, and adolescent psychology, a group counseling program was developed based on the cognitive-behavior theory. The CBGC consisted of three sessions, totaling 10 activities, with each weekly activity lasting 60 minutes. The intervention was delivered to the intervention group for 10 weeks, with a follow-up period of 4 weeks after the end of the intervention. Each session began with a summary and review of the previous session, identified and rated current moods, and shared completed homework and feedback. The session concluded with a summary of the content covered and a homework assignment. All activities were performed by a trained counselor at the university counseling clinic (Table 1).

**Table 1:** Summary of the cognitive-behavioral group counseling program

**Session/** **Activities**	**Topics**	**Objectives**
1/1	Building relationships	To develop the counselee-counselor relationship, introduce the CBGC program, and implement the regulations.
1/2	Psychoeducation: Obesity and WBI	To offer the members the opportunity to practice analysis of obesity, weight stereotypes, and WBI.
1/3	Cognitive-behavioral concepts	To introduce members to cognitive-behavioral concepts for how thoughts, feelings, and behavior interact.
1/4	Identifying the counselee’s problem	To identify and challenge weight-related myths and stereotypes.
2/1	Identifying negative automatic thoughts	To offer members information about how to identify, analyze, and change negative automatic thoughts about oneself due to weight and body size.
2/2	Recording negative automatic thoughts	To inform members about ways to record negative automatic thoughts in response to exposure to weight stigma.
3/1	Introduced to cognitive and behavioral techniques	To offer members information about cognitive and behavioral techniques used to change negative thoughts.
3/2	Transforming negative, automatic thoughts	To offer the members opportunities to find ways to change negative thoughts related to weight by eliciting negative automatic thoughts or reducing self-criticism.
3/3	Problem-solving and coping skills	To equip the members with essential skills to find solutions to problems and respond to WBI.
3/4	Increasing self-efficacy and self-acceptance	To motivate members to change their health behaviors by discussing self-efficacy and self-acceptance, both in general and with respect to achieving health behavior goals.

*Note*. CBGC: Cognitive-behavioral group counseling; WBI: Weight bias internalization.

 The first session included activities such as (1) developing the counselee–counselor relationship and introducing the CBGC program and (2) psychoeducation about obesity, WBI, and stereotypes about weight. The other activities were (3) introducing participants to cognitive-behavioral concepts as a model for how thoughts, feelings, and behavior interact and (4) identifying the counselees’ problems with weight-related myths and stereotypes and determining the problem-solving objectives and goals. Participants were encouraged to share their experiences of weight stigma and its effects with group members. For homework, they were to notice the link between thoughts, feelings, and behavior related to their weight and body size.

 The second session focused on weight-related cognitive restructuring. It consisted of two activities. Participants were instructed on negative automatic thoughts and how to identify, analyze, and change them. Moreover, they practiced thought records and identified their negative automatic thoughts related to weight, including weight stigma. For homework, participants identified negative automatic thoughts from exposure to WBI.

 The third session focused on how to change negative thoughts about oneself due to weight and responses to WBI. It included four activities. Participants were introduced to cognitive and behavioral techniques. In addition, they were shown how to change negative thoughts related to weight by eliciting negative automatic thoughts or reducing self-criticism. Further, the group learned how to respond to WBI by identifying and rating moods when exposed to a weight stigma situation and discussing the right approach. Finally, the participants were motivated to change their health behaviors, increase self-efficacy, and simultaneously practice self-acceptance of their weight and body.

 The follow-up period was 4 weeks. A 5-minute telephone consultation for the intervention group was performed once weekly for 3 weeks to maintain the effect of the intervention, and at week 4, a clinic visit was made for outcome measurements.

###  Control group

 The students allocated to the control group did not receive any of the CBGC program components. Instead, they continued with their usual counseling process, which was offered by the psychologist and did not focus on weight stigma.

###  Study procedure

 Before the CBGC program intervention, the participants received information on the research and its voluntary nature and a declaration of anonymity and confidentiality. All participants then provided written informed consent, and the baseline characteristic and outcome measurements were completed by both groups in a private room. The participants were then randomly assigned to either the control or intervention group. The CBGC program was delivered weekly to the intervention group over 10 weeks. The first session with three activities was delivered for weeks 1–3, the second session with two activities was implemented in weeks 4–5, and the third session with four activities was delivered for weeks 6–10. The follow-up period was 4 weeks for participants in the intervention group, but those in the control group did not receive anything. The outcome measurements were conducted in both groups after intervention completion (at week 10) and at follow-up visits (at week 14). The data collection procedure was the same for both the intervention and control groups. The trained outcome assessors were blinded to the participant allocation.

###  Data analysis

 Descriptive statistics were used to analyze the participants’ characteristics and outcomes. For the comparison of baseline characteristics between the intervention and control groups, chi-square tests and independent-samples t-tests were utilized for categorical variables and normally distributed continuous variables, respectively. The Mann–Whitney U test was applied for skewed continuous variables. The outcome measurements before the intervention, after intervention completion, and at follow-up visits underwent a comparison. The repeated-measure analysis of variance was employed to compare within-group changes, and an independent samples t-test was used for comparison between the intervention and control groups. Additionally, a multivariate linear regression model was performed to determine the intervention effect, adjusting for the baseline characteristics and values of the outcome measures. All statistical analyses were performed using SPSS, version 20.0 (IBM Corp., Armonk, NY, USA). A *P* value of < 0.05 was considered statistically significant.

## Results

 Overall, 80 obese students were enrolled at the baseline and participated until the end of the 4-week follow-up. Most participants were female (62.5%); their median age was 20 years. Approximately 65.0% had a monthly household income of 8000 Thai baht or above (250 US$), 38.8% reported peers with obesity, and 62.5% felt dissatisfied with their body image. The median (interquartile range: IOR) baseline BMI and experience of weight stigma were 27.4 (4.0) and 3.1 (0.6), respectively. The baseline characteristics showed no statistically significant difference between the intervention and control groups (Table 2).

**Table 2:** Baseline characteristics of participants

**Characteristics**	**Total**		**Intervention**		**Control**		***P*** ** value**
	**Number**	**Percent**	**Number**	**Percent**	**Number**	**Percent**	
Age (y)							0.822
< 20	36	45.0	17	42.5	19	47.5	
≥ 20	44	55.0	23	57.5	21	52.5	
Gender							0.817
Female	50	62.5	24	60.0	26	65.0	
Male	30	37.5	16	40.0	14	35.0	
Monthly household income (THB)							0.815
< 8000	28	35.0	15	37.5	13	32.5	
≥ 8000	52	65.0	25	62.5	27	67.5	
Peers with obesity							0.646
Yes	31	38.8	14	35.0	17	42.5	
No	49	61.2	26	65.0	23	57.5	
Body image satisfaction							0.488
Satisfied	30	37.5	13	32.5	17	42.5	
Dissatisfied	50	62.5	27	67.5	23	57.5	
	**Median**	**IQR**	**Median**	**IQR**	**Median**	**IQR**	***P *** **value**
Body mass index (kg/m^2^)	27.4	4.0	27.3	3.9	28.0	4.0	0.790
Experience of weight stigma	3.1	0.6	3.2	0.2	3.1	0.7	0.305

*Note*. THB: Thai baht; IQR: Interquartile range.

 The mean scores of WBI, depression, perceived stress, and self-esteem before the intervention demonstrated no statistically significant differences (*P*> 0.05) between the two groups. After the intervention and follow-up visit, the mean difference between the two groups was statistically significant (*P*< 0.001) for WBI, depression, perceived stress, and self-esteem (Table 3). Based on the comparison of outcomes within groups from before the intervention to the follow-up visit, the mean scores of WBI, depression, and perceived stress in the intervention group decreased significantly (*P*< 0.001), whereas self-esteem increased significantly (*P*< 0.001). However, no significant changes were observed in the control group (Table 2). After adjusting for the baseline characteristics and outcome values, the intervention group showed significant improvements in the scores of WBI (B = -6.82, *P*< 0.001), depression (B = -4.17, *P*< 0.001), perceived stress (B = -6.01, *P*< 0.001), and self-esteem (B = 5.07, *P*< 0.001) compared to the controls (Table 4).

**Table 3:** Comparison of the mean scores of outcome measurements in the intervention and control groups at baseline, after intervention, and follow-ups

**Outcomes/Groups**	**Before intervention**		**After intervention**		**Follow-up**		***P *** **value**
	**Mean**	**SD**	**Mean**	**SD**	**Mean**	**SD**	
Weight bias internalization	31.25	3.74					
Intervention	31.08	4.29	27.58	4.16	23.85	4.76	< 0.001
Control	31.25	3.74	31.05	4.03	30.93	4.02	0.063
Mean difference	-0.17		-3.47		-7.08		
*P *value	0.846		< 0.001		< 0.001		
Depression							
Intervention	22.95	2.49	20.58	2.21	18.50	2.22	< 0.001
Control	23.28	2.24	23.08	2.26	22.90	2.11	0.081
Mean difference	-0.33		-2.50		-4.40		
*P *value	0.541		< 0.001		< 0.001		
Perceived stress							
Intervention	25.83	3.59	22.75	3.40	19.60	2.28	< 0.001
Control	26.65	3.61	26.50	3.56	26.25	3.58	0.082
Mean difference	-0.82		-3.75		-6.65		
*P *value	0.309		< 0.001		< 0.001		
Self-esteem							
Intervention	19.53	2.86	22.75	2.09	24.70	1.89	< 0.001
Control	19.38	2.88	19.53	3.00	19.63	2.89	0.171
Mean difference	0.15		3.22		5.07		
*P *value	0.816		< 0.001		< 0.001		

*Note*. SD: Standard deviation.

**Table 4:** Intervention effects on primary and secondary outcomes between intervention and control groups at the follow-up period

**Outcomes**	**B**^a^	**95% CI**	***P *** **value**
Primary outcome			
WBI	-6.82	-8.06, -5.57	< 0.001
Secondary outcomes			
Depression	-4.17	-4.89, -3.45	< 0.001
Perceived stress	-6.01	-6.79, -5.23	< 0.001
Self-esteem	5.07	4.21, 5.93	< 0.001

*Note*. ^a^ Multivariate regression model adjusted for age, gender, monthly household income, peers with obesity, body image satisfaction, body mass index, and experiences of weight stigma, and baseline values of the outcome measures; CI: Confidence interval; WBI: Weight bias internalization.

## Discussion

 The results of this trial revealed that a CBGC was effective in treating obese youths with WBI and its negative psychological outcomes such as depression, perceived stress, and self-esteem. The cognitive model of self-stigma suggests that the reduction of self-stigmatized beliefs is related to an increase in self-esteem and a decrease in depression and stress.^28,29^ The results support our hypothesis that CBGC would significantly reduce WBI, which is consistent with the results of prior research,^12,13^ reporting that the GCB approach was effective in reducing WBI in individuals with obesity. One possible explanation is that CBGC emphasizes helping youths replace self-stigmatized beliefs with positive beliefs about themselves and recognize their strengths to enhance self-acceptance and self-esteem.^14,30^ Additionally, group interaction activities in the CBGC can improve social support among group members because participants share their specific stigmatized-weight situations and learn to address these through group interaction, which can enhance a sense of self-worth, encourage the sharing of resources and skills to respond to stigma, and lead to a decrease in WBI.^16,30,31^

 Our study also showed that CBGC was effective in decreasing depressive symptoms, which is in line with the findings of previous studies,^30,31^ confirming that GCB significantly improved depression. A possible explanation is that the components of CBGC, including cognitive restructuring, negative thought identification, and self-criticism reduction, which can alter irrational self-stigmatized beliefs and replace them with more positive and rational beliefs,^12,30,31^ can also help participants challenge dysfunctional and irrational beliefs related to their depression symptoms.^32^ CBGC could thus help obese youth decrease their depressive symptoms. Moreover, the group interaction may have been effective in reducing depression because it reduced the participants’ feelings of isolation, helplessness, and loneliness and helped them recognize that they did not have to fight through their struggles alone.^16,18^ The findings of this study also indicated that CBGC significantly reduced stress. In prior studies,^33,34^ GCB had a great effect on stress and increased the use of coping strategies among adolescents. One possible explanation is that CBGC helps adolescents reduce their stress by identifying and changing the negative thoughts and feelings that affect their behavior and avoiding them with coping strategies.^16,33^ Additionally, CBGC can promote coping strategies and personal emotion-management skills to moderate the stress response. Youth also learned to adopt coping mechanisms and adaptability to manage and mitigate stress.^17,33,35^ Additionally, CBGC showed a significant improvement in self-esteem, which conforms to the results of prior studies,^36,37^ indicating that GCB could increase adolescents’ self-esteem. This may be because the CBGC can lead to emotional and behavioral changes, as well as improve self-esteem, by changing maladaptive thoughts and correcting fundamental misconceptions. It can also help youths accept their shortcomings, believe in their competence, dare to appear confidently, and have a sense of self-worth.^38,39^

 This study had some limitations. First, the sample was limited to obese youths from one university counseling clinic in the northeastern region. Thus, generalization of the results to obese youth in other settings might be limited, but it may reflect the situation of obese youth with weight and body size problems in the university-based context. Therefore, further studies may include participants from many settings. Second, our participants were only obese youth who received counseling services. Consequently, they might have had increased motivation to succeed compared to youth who did not seek treatment (e.g., obese youths in the community who have never received treatment or intervention). Hence, caution must be exercised when coming to the results for other groups, and further studies are needed to determine whether the same CBGC program can work in other settings. Third, our subjects were both male and female obese youths, and there was no observed gender difference in WBI and its psychological outcomes, but some previous studies indicated that females have a higher level of WBI than males.^4,8^ Thus, further studies may consider interventions for addressing gender differences in WBI. Fourth, our CBGC program focused mainly on individual-based behaviors to change individuals’ thoughts and feelings related to weight to achieve health behavior goals. However, some studies have indicated that WBI occurs in the context of experiencing stigma through other environmental sources such as media, family, school, work, institutional structures, and social or cultural beliefs.^12,40^ Hence, further studies may need to consider these environmental factors when designing interventions. Despite the limitations, our findings confirmed that the CBGC program is a feasible enhanced intervention that can reduce WBI and its negative psychological outcomes. It can also provide a new counseling method for improving the psychological consequences of obesity in youth. Accordingly, health providers or health counselors should consider applying the CBGC program to improve the WBI and psychological distress of their obese youth clients. Further studies are needed to determine whether reducing WBI may lead to improved physical outcomes such as eating behavior and physical activities.

**Highlights**

- A CBGC program is effective in reducing WBI among obese youths.
- CBGC programs could decrease depression and perceived stress scores but increase the self-esteem score.
- Cognitive-behavioral is a technique for improving psychological outcomes.

## Conclusion

 The CBGC program demonstrated its effectiveness in reducing WBI, depression, and perceived stress while increasing self-esteem among obese youths. Thus, this study suggests that this program should be implemented to reduce WBI and psychological distress in obese youth who have experienced WBI. It should also be integrated into the counseling processes or services provided for youth.

## Acknowledgements

 We are grateful to the Faculty of Public Health, Mahasarakham University, for funding, and we would like to sincerely thank all subjects for their participation.

## Authors’ Contribution

**Conceptualization:** Suneerat Yangyuen, Thidarat Somdee, Atchara Chaichan.

**Data curation:** Suneerat Yangyuen, Meihua Yin, Atchara Chaichan, Supattra Keawmuang.

**Formal analysis:** Suneerat Yangyuen, Atchara Chaichan, Supattra Keawmuang.

**Funding acquisition:** Suneerat Yangyuen, Thidarat Somdee, Supattra Keawmuang.

**Investigation:** Meihua Yin, Thidarat Somdee, Atchara Chaichan, Supattra Keawmuang.

**Methodology:** Suneerat Yangyuen, Atchara Chaichan.

**Project administration:** Suneerat Yangyuen, Thidarat Somdee.

**Resources:** Meihua Yin, Thidarat Somdee, Supattra Keawmuang.

**Software:** Suneerat Yangyuen, Atchara Chaichan, and Supattra Keawmuang.

**Supervision:** Suneerat Yangyuen, Thidarat Somdee.

**Validation:** Suneerat Yangyuen, Thidarat Somdee, Atchara Chaichan.

**Visualization:** Thidarat Somdee, Supattra Keawmuang.

**Writing–original draft:** Suneerat Yangyuen, Atchara Chaichan, Supattra Keawmuang.

**Writing–review & editing:** Suneerat Yangyuen, Meihua Yin, Thidarat Somdee, Atchara Chaichan, Supattra Keawmuang.

## Competing Interests

 The authors declared no conflict of interests.

## Ethical Approval

 This study was reviewed and approved by the Review Ethics Boards of Mahasarakham University (Ref. No. 418-428/2022). Written informed consent was obtained from the participants following the research information, and then the participants were interviewed for baseline data gathering.

## Funding

 This research project was financially supported by the Faculty of Public Health, at Mahasarakham University.
